# YAP1 alleviates sepsis-induced acute lung injury *via* inhibiting ferritinophagy-mediated ferroptosis

**DOI:** 10.3389/fimmu.2022.884362

**Published:** 2022-08-01

**Authors:** Jing Zhang, Yongping Zheng, Yun Wang, Jin Wang, Aming Sang, Xuemin Song, Xinyi Li

**Affiliations:** ^1^ Department of Anesthesiology, Zhongnan Hospital of Wuhan University, Wuhan, China; ^2^ Research Centre of Anesthesiology and Critical Care Medicine, Department of Anesthesiology, Zhongnan Hospital of Wuhan University, Wuhan, China

**Keywords:** sepsis-induced acute lung injury, YAP1, NCOA4, ferritinophagy, ferroptosis

## Abstract

Ferroptosis is a phospholipid peroxidation-mediated and iron-dependent cell death form, involved in sepsis-induced organ injury and other lung diseases. Yes-associated protein 1 (YAP1), a key regulator of the Hippo signaling pathway, could target multiple ferroptosis regulators. Herein, this study aimed to explore the involvement of ferroptosis in the etiopathogenesis of sepsis-induced acute lung injury (ALI) and demonstrate that YAP1 could disrupt ferritinophagy and moderate sepsis-induced ALI. Cecal ligation and puncture (CLP) models were constructed in wild-type (WT) and pulmonary epithelium-conditional knockout (YAP1^f/f^) mice to induce ALI, while MLE-12 cells with or without YAP1 overexpression were stimulated by lipopolysaccharide (LPS) *in vitro*. *In-vivo* modes showed that YAP1 knockout aggravated CLP-induced ALI and also accelerated pulmonary ferroptosis, as presented by the downregulated expression of GPX4, FTH1, and SLC7A11, along with the upregulated expression of SFXN1 and NCOA4. Transcriptome research identified these key genes and ferroptosis pathways involved in sepsis-induced ALI. *In-vitro* modes consistently verified that YAP1 deficiency boosted the ferrous iron accumulation and mitochondrial dysfunction in response to LPS. Furthermore, the co-IP assay revealed that YAP1 overexpression could prevent the degradation of ferritin to a mass of Fe^2+^ (ferritinophagy) *via* disrupting the NCOA4–FTH1 interaction, which blocked the transport of cytoplasmic Fe^2+^ into the mitochondria *via* the mitochondrial membrane protein (SFXN1), further reducing the generation of mitochondrial ROS. Therefore, these findings revealed that YAP1 could inhibit ferroptosis in a ferritinophagy-mediated manner, thus alleviating sepsis-induced ALI, which may provide a new approach to the therapeutic orientation for sepsis-induced ALI.

## Introduction

Sepsis is acute multiple organ dysfunctions induced by the complicated response of the host to intruding pathogenic microorganisms (1, 2), results in expensive hospitalization, and is primarily responsible for hospital mortality (3, 4). The acute systemic inflammatory response arising from sepsis contributes to a cascade of pathological and physiological changes that first spreads to the respiratory system. The lung is especially susceptible to sepsis, which is mediated by inflammation and oxidative stress, leading to acute respiratory distress syndrome or acute lung injury (ARDS/ALI). Sepsis is the dominant cause of ARDS/ALI (6%–42%) (5). ARDS/ALI chiefly manifests as acute inflammation, disruption of endothelial barrier integrity, and injury of the alveolar epithelium, which lead to protein-rich pulmonary interstitial edema and leakage of immune cells into the alveolar cavities (6, 7). Facilitating lung repair and promoting the resolution of lung inflammation are potential therapeutic strategies for ARDS/ALI (7).

Yes-associated protein 1 (YAP1; also regarded as YAP) and transcriptional coactivator TAZ are the essential downstream effectors of the Hippo pathway, which modulates organ development and cell proliferation. The Hippo/YAP1 pathway is a kinase cascade, and YAP1 can be directly phosphorylated and inactivated by the large tumor suppressor 1/2 (LATS1/2), leading to the reservation of YAP1 in the cytoplasm (8). YAP1 could affect the production of autophagosomes and regulate autophagy (9), which are crucial elements of ferritinophagy (10). Previous studies have shown diffuse expression of YAP1/TAZ in multiple tissues, airway and bronchial smooth muscle cells, fibroblasts, and epithelial cells of lung tissue (11–13). YAP1/TAZ expression dysregulation diminished surfactant protein C (SPC) generation, which is a representative element of ARDS (14), and the interaction of TAZ with TTF-1 regulated the activation of SPC in pulmonary epithelial cells (15). Collectively, YAP1/TAZ could bring about lung organogenesis by precisely controlling cell differentiation and proliferation. However, how YAP1 regulates lung epithelial cell proliferation is still indistinct.

As an innovative form of regulated cell death, ferroptosis is described as an iron-dependent phospholipid peroxidation in consequence of reactive oxygen species (ROS) production due to the iron-mediated Fenton reaction (16). Recombinant solute carrier family 7 member 11 (SLC7A11), glutathione (GSH), and glutathione peroxidase 4 (GPX4) are pivotal regulators of the ferroptosis pathway (17). Reductions in GPX4 and GSH levels can be observed during ferroptosis, leading to lipid peroxidation (17, 18). Ferroptosis is mainly regulated by iron homeostasis and oxidative stress, and iron homeostasis is partly controlled by ferritin. Ferritin consists of ferritin light chain (FTL) and ferritin heavy chain 1 (FTH1), the latter being the primary iron-storage protein. Ferritin could be degraded by autophagy (19), which was mediated by nuclear receptor coactivator 4 (NCOA4)—a selective cargo receptor of ferritin. NCOA4-dependent autophagy was defined as ferritinophagy and resulted in increased intracellular iron levels and Fenton reaction (10). One recent research elucidated that the degradation of ferritin was required for ferroptosis cell death of vascular endothelial cells exposed to zinc oxide nanoparticles (20). Bronchial epithelial cell ferroptosis induced by cigarette smoke was closely linked to NCOA4-mediated ferritinophagy (21). Nevertheless, the connection between NCOA4-mediated ferritinophagy and sepsis-induced ALI remains unclear.

Furthermore, the positive effect of YAP1 on pulmonary epithelial cells has already been reported. Research on the intervention of YAP1 in ferroptosis springs up gradually, and the newest study found that YAP depletion could abolish the myocardial protective effect of melatonin by upregulating acyl-CoA synthetase long-chain family member 4 (ACSL4) expression (22). Based on these reports, our study aimed to investigate whether YAP1 was involved in pulmonary epithelial cell ferroptosis in response to LPS stimulation and to explore the underlying mechanism of ferroptosis in sepsis-induced ALI.

## Materials and methods

### Mice

Male mice (8–12 weeks) were used in this experiment. All mice were on the C57BL/6 background, which were obtained from Cyagen (Suzhou, China). YAP1^flox/flox^ mice were crossbred with Sftpc-CreERT2 mice to generate epithelial-specific YAP1-conditional knockout mice (termed as YAP1^f/f^). To obtain epithelial-specific YAP1-conditional knockout mice, 6- to 8-week-old mice received tamoxifen (75 mg/kg dissolved in corn oil, once a day) by intraperitoneal injection for 5 days and had a rest for 4 weeks. Then, we successfully obtained the epithelial-specific YAP1-conditional knockout mice. We used only YAP1^flox/flox^ mice as wild-type mice (termed as WT), and they were used as controls. WT mice and YAP1-knockout mice were randomly allotted into the WT+Sham, WT+CLP, YAP1^f/f^+Sham, and YAP1^f/f^+CLP groups. All experiments were executed following the criteria of the NIH and authorized by the Animal Ethics Committee of Wuhan University (No. 2021187).

### Cecal ligation puncture in mice

The sepsis-associated lung injury model mice were created by cecal ligation and puncture (CLP) according to previous studies (23). After using 50 mg/kg of pentobarbital sodium (1%, Sigma, USA) to anesthetize the experimental mice, about a 1-cm midline incision was operated in the mice’s lower abdomen. The procedure was made to dissociate and ligate the cecum, which was punctured with a 20‐gauge needle twice. After gently squeezing the cecum to push the feces into the abdominal cavity, the cecum was anastomosed and the abdomen was sutured layer by layer. Volumetric resuscitation was performed immediately after surgery with 25 ml/kg of Ringer saline in mice. For the mice in the sham group, the cecum was just turned over when the abdomen was opened, and no treatment was given as mentioned above except closing the abdomen. After the treatment, the mice were administered 1 ml of warming sterile saline for fluid resuscitation immediately. The mice enjoyed free access to food and water and were monitored until sacrificed 24 h after the operation.

### Cell models

MLE-12 cells (1 × 10^5^ cells, provided by the ATCCATCC, USA) were cultured with 100 U/ml of streptomycin/penicillin and fetal bovine serum (10%) in DMEM (Gibco, USA) and arranged in an incubator containing 5% CO_2_ with a suitable temperature (37°C). Cells were seeded in 24-well plates. To overexpress a certain gene—*YAP1*, the YAP1 overexpression clone lentiviral particle (YAP1 OE) of MLE-12 cells was supplied by GeneChem Co. Ltd. (P40122, Shanghai, China). For the stable overexpression of the *YAP1* gene, the cells were cultured in 24-well plates. Transfection of *YAP1* was accomplished referring to the working instructions, and cells transfected with scramble were used as controls. Both scramble and YAP1 OE were transfected into the MLE-12 cells for 48 h before LPS stimulation. Following expansion and maintenance, stable MLE-12 cells expressing YAP1 overexpression were used for the subsequent experiments. The transfection efficiency was detected by Western blot, and the nuclear translocation of YAP1 was captured by fluorescence microscopy. To generate pulmonary injury models *in vitro*, LPS (1 μg/ml, Sigma, USA) was given to the scramble or YAP1 overexpression cells for 24 h (**Figures S1A–D**). MLE-12 cells were randomly allotted into the control group (Con+Scramble), LPS group (LPS+Scramble), control+YAP1 overexpression-treated group (Con+YAP1 OE), and LPS+YAP1 overexpression-treated group (LPS+YAP1 OE).

### Determination of protein content in bronchoalveolar lavage fluid

Bronchoalveolar lavage fluid (BALF) was obtained from the left lung post-experiment. Abiding with the manufacturer’s protocols, after euthanizing the mice, they were fixed, the skin was incised in the middle of the neck, the trachea was isolated, and the right bronchus was ligated. The left bronchus was punctured by a fine needle and fixed with silk thread. Then, 0.5 ml of PBS solution was injected into the bronchus, and the BALF was recovered slowly after 1 min. The above operation was repeated five times. The collected BALF was centrifuged 1,500 rpm at 4°C for 10 min, and the supernatant was recovered and stored at −20°C for protein concentration determination. The content of protein was checked by the commercial bicinchoninic acid protein assay kit (Beyotime, China).

### Histological analysis

The lung samples were isolated and fixed with 4% paraformaldehyde for 48 h and then dehydrated by ethanol solution with different concentrations (70%–100%) for 40 min. Following paraffin embedding, the samples were preserved for the subsequent experiments. The sections were sliced into 4 μm sections and placed in xylene, anhydrous ethanol, and alcohol to dewax in sequence. Hematoxylin–eosin (HE) was utilized to stain the lung samples. The slides were dyed with hematoxylin for 5 min and rinsed with water, and then after returning to blue, the slides were stained with eosin for 1–3 min. The degree of lung damage was assessed by two independent technicians who were blinded to the experimental group protocols in line with the recently published criterion (24). The scores were recorded according to the degree of neutrophil infiltration, alveolar and interstitial edema, and hemorrhage in lung tissues. The range of scores was from 0 to 4: 0, normal lung; 1, mild lung injury (less than 25% injury); 2, moderate lung injury (25%–50% injury); 3, broad lung injury (50%–75% injury); and 4, extreme lung injury (more than 75% injury). The individual scores of each criterion were added and calculated as the final lung injury score. The histological photographs were observed by optical microscopy (Nikon, Japan).

### Immunohistochemical stain*ing*

Lung tissues were paraffin-fixed and incubated overnight at 37°C. Then, these lung sections were deparaffinized and hatched with 3% hydrogen peroxide for 15 min. The slices were heated at microwave treatment and then naturally cooled for 40 min. After performing antigen retrieval, the samples were blocked with 1% BSA for 0.5 h and incubated with primary rabbit anti-NCOA4 (1:100, DF4255, Affinity Biosciences, Jiangsu, China) and anti-SLC7A11 (1:100, DF12509, Affinity Biosciences, Jiangsu, China), respectively, overnight at 4°C. Lastly, homologous fluorescent or biotin-labeled secondary antibodies were incubated with the lung tissues for 2 h at 37°C. The images were captured using a microscope (Nikon, Japan).

### Cell viability assay

Following the previous illustration, cell viability was examined by the CCK-8 assay kit (Beyotime, China) (25). The CCK-8 solution (10 μl) was diluted into the working concentration and given into each well of MLE-12 cells and cultured with cells. After 3 h, the microplate reader (PerkinElmer, USA) was used to analyze the absorbance (450 nm).

### Transmission electron microscopy

The fresh lung lobe was removed and placed in a fixed solution quickly. Briefly, about 1-mm^3^ piece of lung sections was fixed in 2.5% glutaraldehyde, and then the samples were fixed with 1% osmium tetroxide for 90 min. Next, the samples were stained with 2% uranyl acetate and in turn dehydrated with a cascade of ethanol series. The slices were embedded in acetone (100%, 4°C) all night and cut into ultrathin slices (100 nm). Lastly, these slices were double-dyed with lead citrate and uranyl acetate. The graphs were observed using an HT-7500 transmission electron microscope (Hitachi Co., Japan).

### Determination of reactive oxygen species

MLE-12 cells were suspended by 0.25% trypsin and subsequently centrifuged at 1,500 rpm (4°C, 5 min). The cells were cultured in 24-well plates and received the corresponding treatment at the given time according to the indicated groups. The cells were incubated with the 2′,7′-dichlorodihydrofluorescein diacetate (DCFH-DA) fluorescent probe (D6883, Sigma-Aldrich, MO, USA) in a serum-free medium following the manufacturer’s protocols. Shortly, the probe incubation solution should be firstly prepared under dark conditions, and the probe was diluted (1:1,000) to 10 μM of the working concentration. Secondly, the cells were washed with PBS and incubated with a 500-μl probe solution of each well for 15 min at 37°C. Finally, discarding the probe solution and washing it three times, the cells were added with a 400-μl medium. The level of ROS in MLE-12 cells was acquired by a TE-2000 fluorescent microscope (Nikon, Japan) at an excitation (Ex) wavelength of 485 nm and an emission (Em) wavelength of 530 nm.

Lipid ROS was detected by the BODIPY 581/591 C11 reagent (D3861, Invitrogen, California, USA). Briefly, after the indicated treatment, the cells were dyed with a 5-μM reagent and incubated for 30 min. Flow cytometry (BD Accuri C6 Plus, BD Biosciences, USA) and FlowJo software were adopted to detect the lipid ROS level. Quantification of ROS and lipid ROS in individual cells was analyzed and calculated by the software Image-Pro Plus 6.0, (Rockville, USA) and the Con+Scramble group was used for the normalization of the data (regarded as fold change 1).

### Determination of reactive oxygen species in lung tissue

The ROS level of lung tissue was evaluated with the dihydroethidium (DHE) fluorescent probe (D7008, Sigma-Aldrich, USA). The lung tissues were fixed in 4% paraformaldehyde for 48 h and dehydrated in gradient sucrose. Then, the samples were embedded in optimal cutting temperature compound (OCT gel) and serially sectioned to 8 μm. The already frozen sections were incubated with 50 μM DHE away from light at 37°C for 30 min and 1 mg/ml DAPI for 10 min. To obtain the images, we used a Nikon TE-2000 fluorescent microscope (Tokyo, Japan) at (Ex/Em) 525 nm/610 nm. Quantification of ROS in lung tissue was analyzed and calculated by the software Image-Pro Plus 6.0 targeted to the hole images, and the WT+Sham group was used for the normalization of the data (regarded as fold change 1).

### Determination of mitochondria ROS

MLE-12 cells were seeded in 24-well lucifugal plates, then incubated with 5 μM MitoSOX™ reagent solution (Beyotime, China) for 10 min. The cells were washed two times with PBS, and the level of mitochondria ROS was assessed by a fluorescence microscope (TE-2000, Nikon Co., Japan). Quantification of the mitochondrial ROS in individual cells was analyzed and calculated by the software Image-Pro Plus 6.0, and the Con+Scramble group was used for the normalization of the data (regarded as fold change 1).

### Detection of related indicators

The levels of ferrous iron (Fe^2+^), malondialdehyde (MDA), and glutathione (GSH) in MLE-12 cells and lung tissue lysate were detected by the iron assay kit (MAK025, Sigma-Aldrich, USA), MDA assay kit (S0131, Beyotime, China), and GSH assay kit (S0053, Beyotime, China) according to relevant manufacturers’ protocols.

### Western blot

We extracted the lysate from lung tissues or cells in a RIPA buffer, and protein contents were quantified by the BCA protein assay kit (P0011, Beyotime, China). Protein samples (50 μg) from each group underwent the 10% SDS-PAGE gel electrophoresis and then transferred to a PVDF membrane. After being blocked with non-fat dry milk (5%), these blots were hatched with the primary antibodies all night at 4°C. The proteins used in the study were as follows: GPX4 (DF6701, Affinity Biosciences, Jiangsu, China), ACSL4 (1:1,000, A14439, ABclonal Technology, Wuhan, China), SFXN1 (1:1,000, DF12509, Proteintech Group, Wuhan, China), LC3 (1:1,000, AF5402, Affinity Biosciences, Jiangsu, China), NCOA4 (1:10,000, DF4255, Affinity Biosciences, Jiangsu, China), FTH1 (1:1,000, DF6278, Affinity Biosciences, Jiangsu, China), SLC7A11 (1:1,000, DF12509, Affinity Biosciences, Jiangsu, China), YAP1 (1:1,000, DF3182, Affinity Biosciences, Jiangsu, China), or β-actin (BM0627, Boster Biological Technology, Wuhan, China). Finally, the proteins were incubated with HRP-conjugated secondary antibody (1:5,000, Proteintech) at room temperature for 1 h. An ECL kit (Beyotime) was applied to detect the bands, which were assayed by the ImageJ software.

### Distribution of ferrous iron in lysosomes

MLE-12 cells were plated in a 96-well culture dish and hatched overnight. After simulating the four groups of cells indicatively, the 50-nM LysoTracker Green (Beyotime, C1047s, China) and 1-μM FerroOrange (Dojindo, F374, Japan) were added to the cells for 30 min to co-stain the cells and incubated at room temperature for 30 min, and then the previous solution was replaced with fresh PBS. After washing with PBS, the samples were incubated with secondary antibodies including rabbit anti-mouse IgG Ab (A11059, Invitrogen), goat anti-rabbit IgG Ab (A-11012, Invitrogen), or DAPI. The cell photographs were captured by a confocal microscope (TCS-SP2, Leica, Germany) to acquire the mitochondrial ferrous iron and green fluorescent lysosomes at Ex and Em wavelengths of 488 and 510–550 nm, respectively. Quantification of the FerroOrange and colocalization in each hole cell were analyzed and calculated by the software Image-Pro Plus 6.0, and the Con+Scramble group was used for the normalization of the data (regarded as fold change 1).

### Ferritin and LAMP2

MLE-12 cells were fixed as pre-described and immunostained by anti-ferritin (Abcam, ab75973, UK) and anti-LAMP2 (Proteintech, 66301-1-Ig, USA). Then, the samples were incubated with secondary antibodies or DAPI. MLE-12 cells were photographed using confocal laser scanning microscopy and fluorescence microscopy (TE-2000, Nikon Co., Tokyo, Japan). Quantification of the ferritin and lysosomes in each hole cell was analyzed and calculated by the software Image-Pro Plus 6.0, and the Con+Scramble group was used for the normalization of the data (regarded as fold change 1).

### Colocalization of LC3 and ferritin

Firstly, MLE-12 cells with stable YAP1 overexpression were transfected with LC3-GFP (Beyotime, D2815, China) and cultured in eight-well plates for 24 h. Secondly, the cells in each group were treated with LPS and bafilomycin A1 (BafA1) (Abcam, ab120497, UK) for 24 h. Thirdly, the cells were permeated by 0.03% Triton X-100 (Beyotime, China) for 60 min and then fixed and blocked with 0.1% BSA (Beyotime, China) for 1 h. Finally, the primary anti-ferritin (Abcam, ab75973, UK) and secondary antibodies were applied to the medium in turn. MLE-12 cells were analyzed *via* confocal laser scanning microscopy and evaluated by fluorescence microscopy (Nikon TE-2000, Tokyo, Japan).

For immunofluorescence staining in lung tissues, the samples were fixed in 4% paraformaldehyde for 1 h, washed completely with PBST (1× PBS added with 0.1% Triton X-100) for three times, and permeabilized with 0.1% Triton X-100 for 1 h. Then, the samples were incubated with primary antibodies including anti-LC3 (1:100, AF5402, Affinity Biosciences) and anti-ferritin (1:100, Abcam, ab75973) for 10 h at 4°C. After three times of washing with PBST, the samples were incubated with secondary antibodies, and nuclei were stained with DAPI. After washing again, the samples were imaged with a confocal microscope (Nikon TE-2000).

### Co-immunoprecipitation assay

Following the corresponding pretreatment in the indicated groups, the concentration of protein in MLE-12 cells was assessed by the BCA protein (Beyotime, Shanghai, China) assay kit. MLE-12 cells were lysed in mixed buffer (50 mmol/L Tris–HCl, 150 mmol/L NaCl, 1% NP-40, 1 mmol/L NaF, 1 mmol/L EDTA, 1 mmol/L PMSF, 1 mmol/L Na_3_VO_4_, and protease inhibitor cocktail). The protein supernatant was hatched with 1–2 μg rabbit polyclonal IgG control antibody and 25 μl resuspended volume of protein A/G plus agarose (Beyotime, P2055) for 1 h. Then, the protein supernatant was incubated with 1 μl anti-NCOA4 (Affinity Biosciences, DF4255, 1:1,000) for 24 h at 4°C. The cells were cultured again lasting 2 h. The immunoprecipitation buffer was washed several times, and 50 μl of SDS-PAGE loading buffer was added and then denatured. Finally, the co-IP assay was performed.

### RNA sequence analysis

First, RNA was extracted from CLP or normal lung tissue. Bioanalyzer 2100 system was used to detect RNA purity. Ribo-off rRNA Depletion Kit (N406-02, Vazyme, China) and MGIEasy RNA Library Prep Kit (1000006385, MGI, China) were used to generate sequencing libraries according to the manufacturer’s instructions. Then, after purifying the PCR products with the AMPure XP system, the library quality was analyzed (Agilent Bioanalyzer 2100 system). Finally, the index codes were clustered by Cluster Kit of HiSeq 4000 PE (Illumina, USA), and the sequenced library preparations were performed for 150 cycles on the former platform. Total library constructions and sequencing were implemented at BGI Wuhan. Hierarchical clustered heatmap and genome enrichment analysis were adopted to detect the differential expression of both groups. Differentially expressed genes (DEGs) were displayed for log2 fold change >1 and adjusted *p*-value <0.05.

### Statistical analysis

All data are presented as the means ± standard deviation and analyzed by SPSS software 23.0. Graphs were drawn by GraphPad Prism 8.0. Discrepancies among the four groups were checked by one-way ANOVA analysis. *p <*0.05 was defined as statistically significant.

## Results

### YAP1 overexpression prevented ferroptosis in LPS-stimulated MLE-12 cells

**Figure 1:**
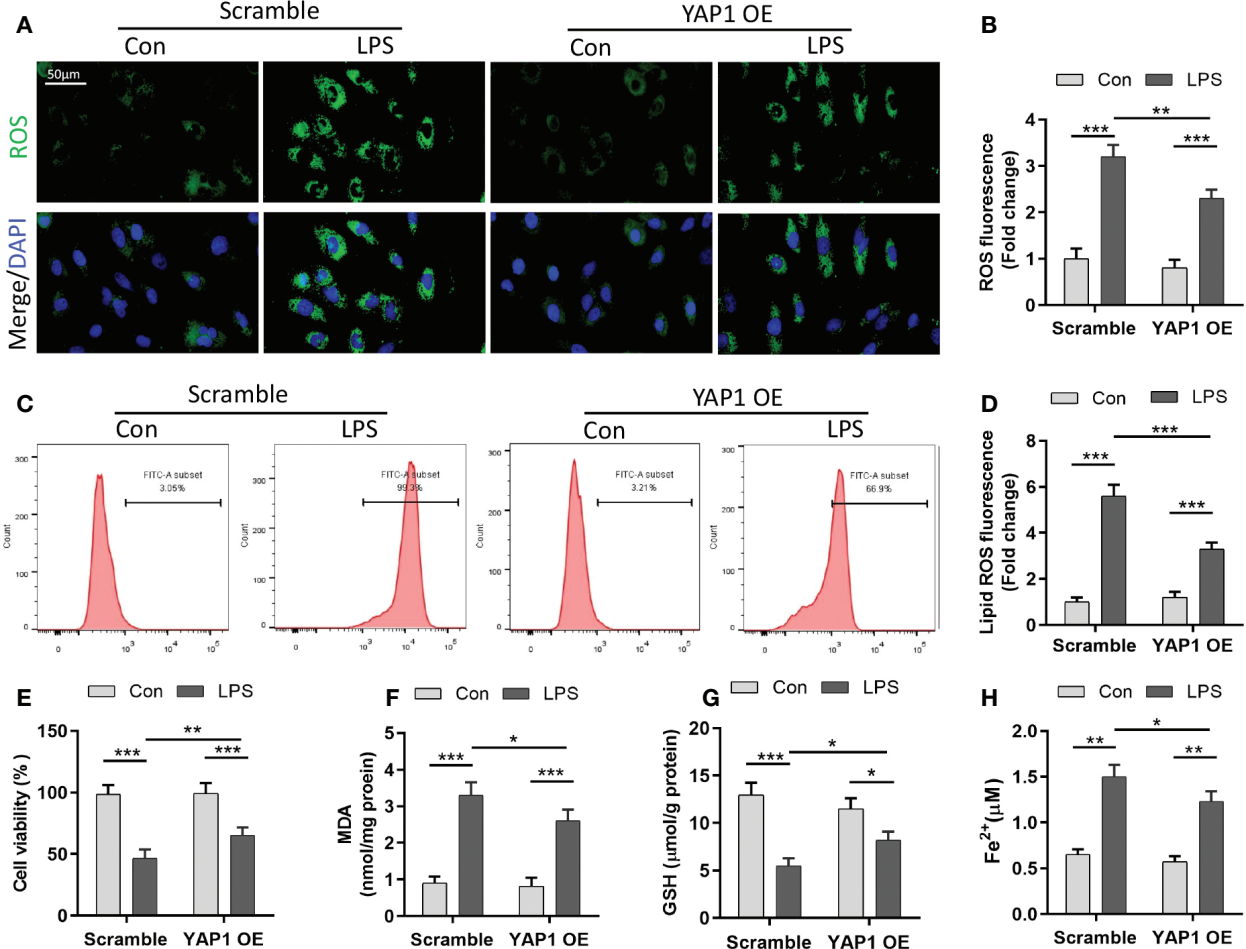
Yes-associated protein 1 (YAP1) alleviated reactive oxygen species (ROS) accumulation and lipid peroxidation in LPS-induced MLE-12 cells. **(A)** Representative images of fluorescence probes (DCFH-DA) for intracellular ROS production and **(B)** quantification of ROS fluorescence intensity. **(C)** Lipid ROS generation was analyzed by using BODIPY 581/591 and determined by flow cytometry in MLE-12 cells treated with the contextual stimuli and **(D)** quantification of lipid ROS. **(E)** Cell viability was evaluated by the cell counting kit-8. The contents of MDA **(F)**, GSH **(G)**, and Fe^2+^ **(H)** were determined using the indicated commercial kits. Data are expressed as mean ± SD. *n* = 3; *p < 0.05, **p < 0.01, ***p < 0.001. LPS, lipopolysaccharide; Con, control group; Scramble, negative control; YAP1 OE, YAP1 overexpression.

We explored that LPS could induce ferroptosis in LPS-treated pulmonary epithelial cells (**Figures S2A–D**). To delineate the presence of ferroptosis in LPS-induced ALI, MLE-12 cells were transfected with YAP1 overexpressing lentivirus (YAP1 OE). First, we detected the verification of YAP1 overexpression (**Figures S3A, B**) and nuclear translocation of YAP1 in MLE-12 cells (**Figures S3C**). Elevated cellular ROS accumulation and lipid peroxidation were not merely the vital origins of ferroptosis but also the hallmarks of ferroptosis. We found that LPS treatment induced remarkable ROS accumulation in MLE-12 cells, which was captured by the enhancement of green fluorescence, while YAP1 overexpression significantly diminished the fluorescence intensity of ROS (**Figures 1A, B** **)**. In line with ROS tendency, abundant accumulation of lipid ROS was observed in LPS-treated cells; however, YAP1 overexpression partially reversed the lipid ROS accumulation induced by LPS from flow cytometry (**Figures 1C, D** **)**. Moreover, LPS distinctly gave rise to the mortality of MLE-12 cells; meanwhile, YAP1 overexpression could increase the number of viable cells upon LPS treatment (**Figure 1E**). The lipid peroxide production (MDA) levels and the content of iron (Fe^2+^) increased (**Figures 1F, H** **)**, whereas the levels of GSH declined (**Figure 1G**) following LPS treatment, both of which are the representative hallmarks of ferroptosis. These aforesaid related markers were converted to beneficial tendency *via* overexpressing YAP1 in MLE-12 cells (**Figures 1F–H**). Altogether, these outcomes suggested that LPS induced ferroptosis, which could be partially counteracted by YAP1 overexpression in MLE-12 cells.

### YAP1 overexpression decreased the amount of intracellular free-divalent iron to inhibit ferroptosis

**Figure 2:**
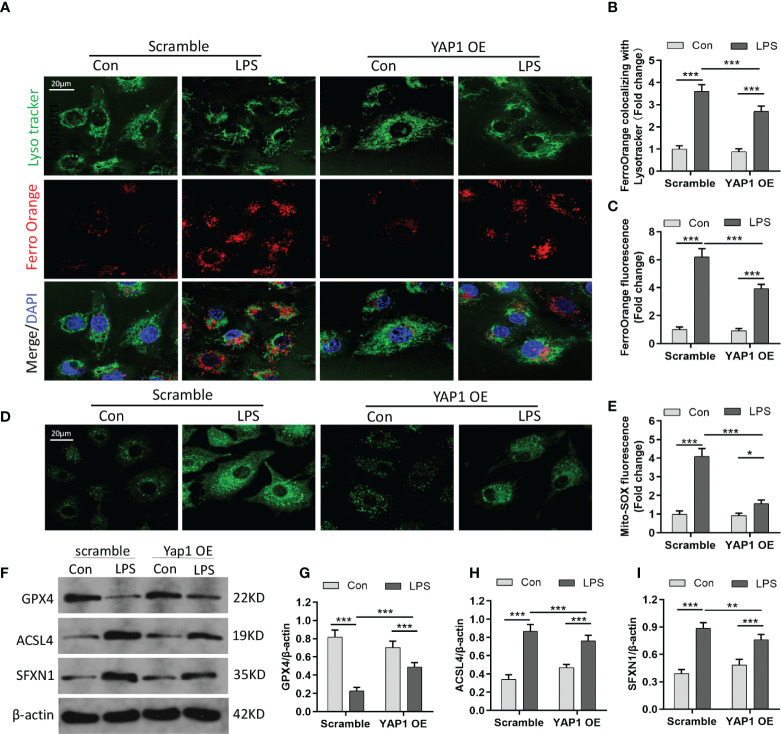
YAP1 activation inhibited ferroptosis by decreasing the amount of intracellular free ferrous iron. **(A)** LysoTracker (green) and FerroOrange (red) were shown by confocal imaging in MLE-12 cells stimulated with LPS. The images exhibited the localization of ferrous iron in survival cells. The lysosomes were stained by LysoTracker Green fluorescence. Nuclei were stained with DAPI (blue). **(B)** Quantification of the fluorescence intensity of FerroOrange colocalized with LysoTracker (green). **(C)** Quantification of the FerroOrange fluorescence intensity. **(D)** Representative images of mitochondrial ROS stained with MitoSOX (green). **(E)** Quantitative results of mitochondrial ROS. **(F–I)** The expression levels of GPX4 **(G)**, ACSL4 **(H)**, and SFXN1 **(I)** were shown by WB. Data are expressed as mean ± SD. *n* = 3; *p < 0.05, **p < 0.01, ***p < 0.001.

Divalent metal ion transporter 1 (SLC11A2) mediates the release of Fe^2+^ into a labile iron pool of cytoplasm. Excessive iron would contribute to the Fenton reaction to produce massive ROS and activate iron-containing enzymes to facilitate lipid peroxidation and ferroptosis. We chose the Fe^2+^-specific probe FerroOrange to explore whether YAP1 could affect the contents of bioavailable Fe^2+^. The level of Fe^2+^ (FerroOrange) remarkably decreased in YAP1 overexpression cells on the basis of the LPS stimuli (**Figures 2A, C** **)**. We also observed that colocalization of LysoTracker (green) and FerroOrange (red) was strengthened after LPS stimulation, indicating that large amounts of iron were present in lysosomes. However, the location of free Fe^2+^ in lysosomes was weakened in YAP1 overexpression cells upon LPS stimulation (**Figures 2A, B** **)**. The number of lysosomes remained the same in each group (**Figures S4A**), illustrating that the decrease in the amount of lysosomal Fe^2+^ was independent of the number of lysosomes.

SFXN1 (as a mitochondrial amino acid transporter) can facilitate the component of iron utilization traversing the mitochondria (26). In the pathological states, the expression of SFXN1 protein was elevated, combined with the enhancement of iron absorption (27). We explored that mitochondrial ROS sharply increased in LPS-induced cells *via* the MitoSOX green fluorescence probe. However, YAP1 overexpression could significantly reduce the mitochondrial ROS accumulation in LPS-treated cells (**Figures 2D, E** **)**. GPX4, which acts as one of the redundant defense mechanisms in the mitochondria, was inhibited after LPS stimulation and turned high level when in alliance with YAP1 overexpression (**Figures 2F, G** **)**. As shown, apart from diminishing the expression level of ACSL4, YAP1 overexpression could inhibit the expression of SFXN1 when cells accepted LPS inducement (**Figures 2F, H, I** **)**. To some extent, YAP1 inhibited ferroptosis by reducing free iron and ROS located in lysosomes.

### YAP1 overexpression blocked the degradation of ferritin in lysosomes (ferritinophagy) and *was* localize*d* in autophagosomes

**Figure 3:**
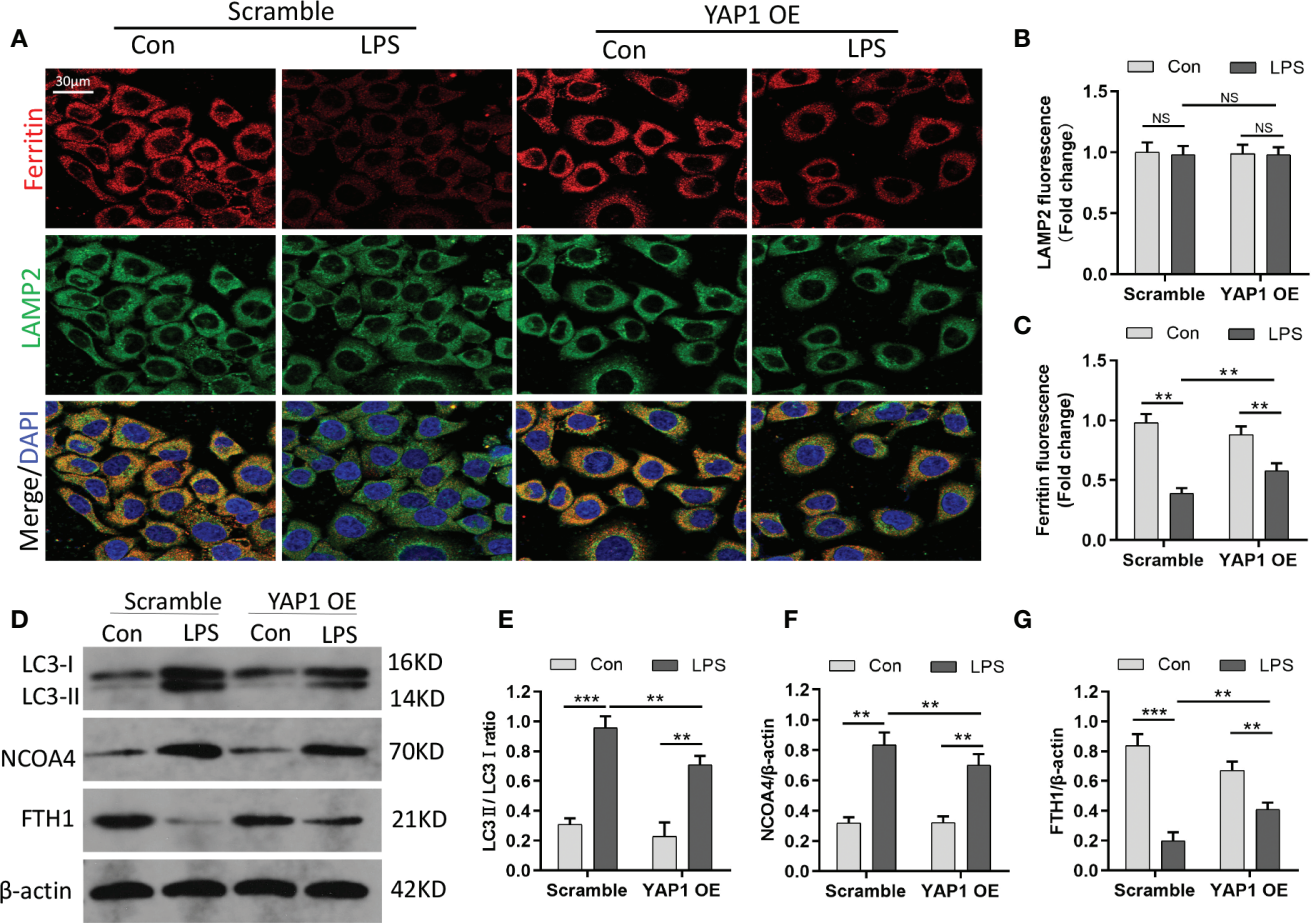
YAP1 restrained the degradation of ferritin in lysosomes to suppress ferritinophagy. **(A)** Confocal images of ferritin (red) and LAMP2 (green) were shown in LPS-treated MLE-12 cells, while lysosomes were stained with LAMP2 (green). **(B)** Quantification measurement of lysosome fluorescence intensity. **(C)** Quantification measurement of ferritin fluorescence intensity. **(D–G)** Data showed the expression contents of LC3II/LC3I **(E)**, NCOA4 **(F)**, and FTH1 **(G)**. Data are expressed as mean ± SD. *n* = 3; *p < 0.05, **p < 0.01, ***p < 0.001. NS: no significance, P>0.05.

Ferritin is the primary iron-storage protein in mammals and a crucial element in maintaining iron homeostasis and preventing Fenton reaction. Ferritin can alter sequestered iron *via* the process of autophagy (ferritinophagy). Since YAP1 overexpression decreased the amount of intracellular free-divalent iron (as shown in **Figure 2A**), we speculated that this process might be linked to ferritinophagy (degradation of ferritin in lysosomes); we thus hypothesized that YAP1 overexpression could interrupt ferritinophagy. To validate the assumption, we carried out immunofluorescence assays to observe the lysosomal location of ferritin. The MLE-12 cells were fixed and immunostained using antibodies of ferritin (red fluorescence) and LAMP2 (green fluorescence). In baseline conditions, the control group with no LPS treatment presented high ferritin fluorescence, indicating a small quantity of degraded ferritin. Surprisingly, upon LPS treatment of the control cells, ferritin fluorescence was substantially decreased due to the degradation of ferritin located in lysosomes. Furthermore, in LPS-stimulated cells, the levels of ferritin were partially restored (YAP1 OE+LPS vs. Scramble+LPS) upon YAP1 overexpression compared to the scramble group (**Figures 3A, C** **)**. The number of lysosomes was unchanged (**Figure 3B**). The results suggested that YAP1 inhibited the lysosomal ferritin degradation upon LPS treatment and led to impaired ferritinophagy after LPS-stimulated injury.

It had been reported that NCOA4 can combine with FTH1 and targeted ferritin to lysosome for autophagy degradation to release free iron. Blocking autophagy or knocking out NCOA4 inhibits the labile accumulation of iron and ROS associated with ferroptosis and prevents eventual iron-related cell death (28). To further investigate ferritinophagy, we determined the levels of LC3, NCOA4, and FTH1 by Western blotting. We found that the expression of the autophagic marker (LC3) and NCOA4 was elevated in MLE-12 cells exposed to LPS, while YAP1 overexpression declined the expression levels on the basis of LPS stimulation (**Figures 3D–F**). Along with the fluorescence images, YAP1 overexpression partially recovered the decreasing levels of FTH1 caused by LPS treatment (**Figures 3D, G** **)**. Overall, YAP1 could inhibit ferritinophagy in LPS-challenged MLE-12 cells.

### YAP1 overexpression inhibited ferritinophagy by disrupting the NCOA4–FTH1 interaction

**Figure 4:**
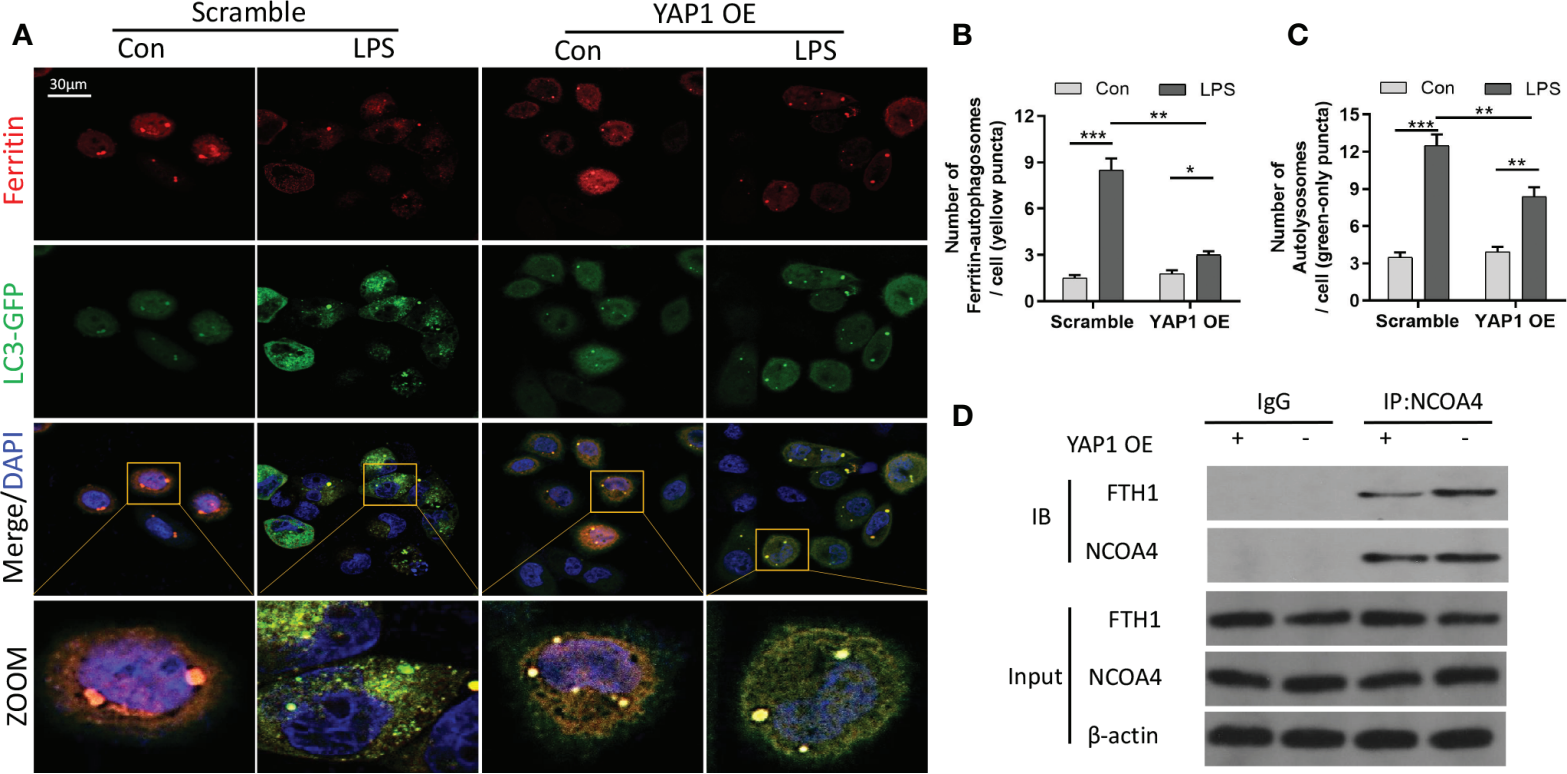
YAP1 inhibited ferritinophagy by disrupting the NCOA4−FTH1 interaction. **(A)** Cells in the indicated groups were transfected with the LC3-GFP plasmid, and the autophagosome in cells presented green fluorescence puncta. Representative confocal images of cells exhibited fluorescent colocalization (yellow puncta) of ferritin (red fluorescence) with LC3-GFP (green puncta). Cells were treated with LPS for 24 (h) **(B)** Fluorescence quantification of ferritin colocalizing with autophagosomes (LC3-GFP). The colocalization puncta per cell were calculated. **(C)** Fluorescence quantification of autophagosomes (LC3-GFP). The autophagosome puncta per cell were calculated. **(D)** Immunoprecipitation analysis of NCOA4 and FTH1 interaction in MLE-12 cells. IgG was used for control. Data are expressed as mean ± SD. *n* = 3; *p < 0.05, **p < 0.01, ***p < 0.001.

Ferritinophagy (autophagic degradation of ferritin) can promote ferroptosis and is mediated by the cargo receptor NCOA4. The immunofluorescence analysis showed that LPS treatment decreased ferritin fluorescence and enhanced the expression of LC3-GFP in MLE-12 cells. The colocalization fluorescence of ferritin with LC3-GFP was strengthened by LPS exposure, whereas YAP1 overexpression reduced the numbers of LC3-GFP puncta (green puncta) and colocalization puncta of ferritin with LC3-GFP (yellow puncta) in the LPS-treated cells (**Figures 4A–C**). To further investigate the interaction of NCOA4-mediated ferritinophagy and FTH1, we performed co-IP assays to detect the inner interaction between NCOA4 and FTH1 in the MLE-12 cells. YAP1 overexpression significantly decreased the association of FTH1 with NCOA4 (**Figure 4D**), suggesting that YAP1 might disrupt the interaction of NCOA4 and FTH1.

Taken together, these outcomes hinted that YAP1 inhibited ferritinophagy by suspending the NCOA4–FTH1 interaction in LPS-treated MLE-12 cells.

### YAP1 inactivation accelerated ferroptosis activation during CLP-induced acute lung injury

**Figure 5:**
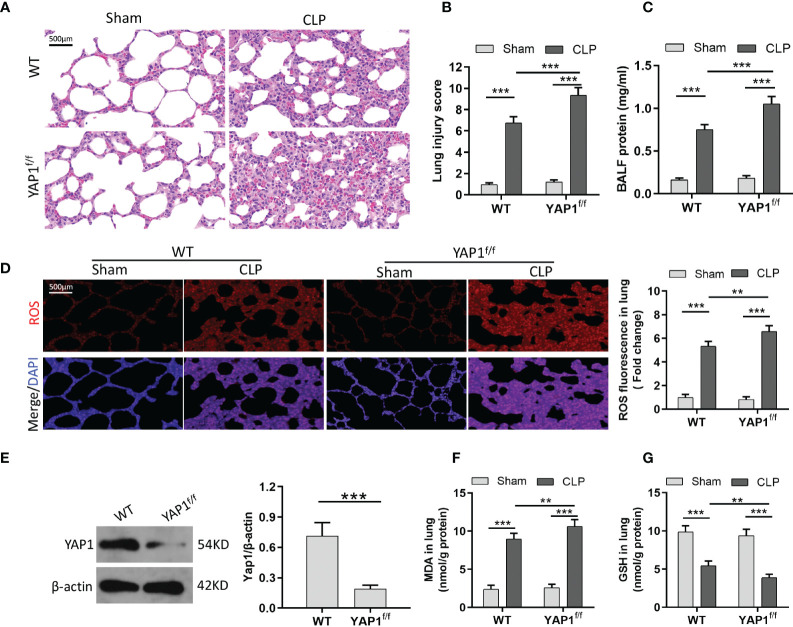
YAP1 deficiency aggravated oxidative stress-mediated lung injury in CLP mice. **(A)** Histological images of lung samples exhibited by HE staining in mice following CLP, YAP1^f/f^, and CLP+YAP1^f/f^ treatment. **(B)** The pathological lesion scores. **(C)** The content of BALF protein. **(D)** Representative fluorescent images of ROS staining and the quantification of ROS fluorescence intensity in lung tissue. **(E)** The certification of YAP1 conditional knockout in mice pulmonary epithelial cells. **(F–G)** Pulmonary content of MDA **(F)** and GSH **(G)** was assessed by the corresponding commercial kit. Data are expressed as mean ± SD. *n* = 6; *p < 0.05, **p < 0.01, ***p < 0.001.

To verify the role of YAP1 with ferroptosis in sepsis-induced ALI *in vivo*, we used the YAP1 conditional knockout mice for further research. As shown, CLP treatment created significant pathological changes, including alveolar hemorrhage and massive inflammatory cell infiltration, while YAP1 conditional knockout mice suffered larger trauma (**Figures 5A, B** **)**. Furthermore, YAP1 deletion exacerbated CLP-stimulated pulmonary edema significantly as evidenced by protein leakage in BALF (**Figure 5C**). These results confirmed that YAP1 was a crucial element in CLP-induced ALI.

Similar to MLE-12 cells, to prove the anti-ferroptotic role of YAP1 in CLP-treated mice, we measured the ROS production and lipid peroxidation-related markers (MDA and GSH) with or without YAP1 deficiency in the murine lung tissue. Western blot data exhibited that the YAP1 protein level was significantly decreased in YAP1^f/f^ mice (**Figure 5E**). CLP treatment markedly increased the generation of ROS and MDA levels and diminished GSH contents, while YAP1 deficiency deteriorated the oxidative damage caused by CLP treatment (**Figures 5D–G**). Collectively, we considered that YAP1 conditional knockout in mice aggravated pulmonary ferroptosis in response to CLP stimulation.

### RNA-seq identified the enriched ferroptosis pathway and upregulated YAP1 in sepsis-induced ALI

**Figure 6:**
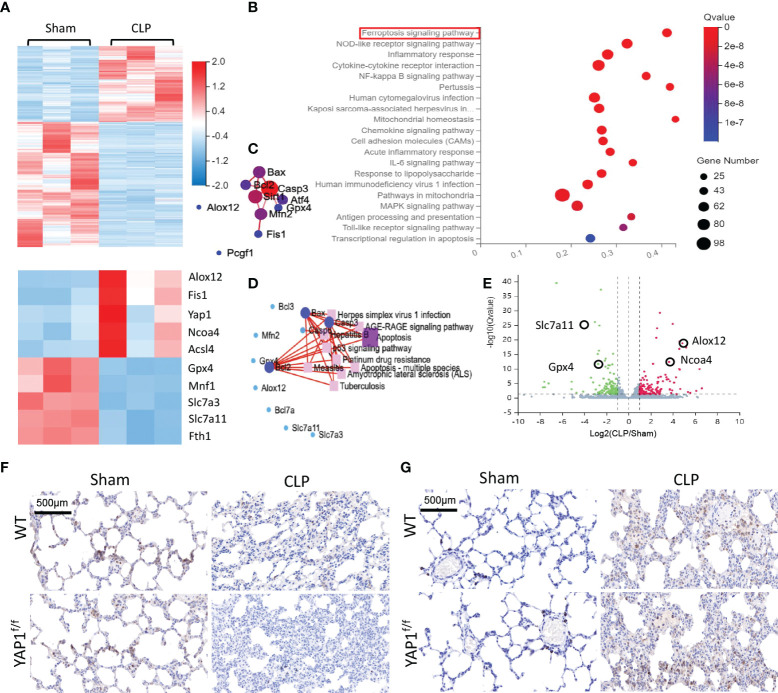
Gene expression analysis of lung tissue in WT+CLP mice and WT+Sham mice. **(A)** Hierarchical clustered heatmap of differentially expressed genes (DEGs) in lung tissues between WT+CLP mice and WT+Sham mice, *n* = 3. **(B)** The Kyoto Encyclopedia of Genes and Genomes enrichment analysis (KEGG pathway) was adopted to identify the most significantly altered signaling pathways in lung tissues of CLP mice. The differentially expressed genes enriched in the ferroptosis-related pathway, indicating that the ferroptosis signaling pathway was obviously activated, *n* = 3. **(C)** Construction of protein–protein interaction (PPI) regulatory network based on DEGs. **(D)** Network of KEGG pathway based on similarity of their gene expression profiles. These enrichment genes were related to several signaling pathways. **(E)** The volcano plot shows DEGs between CLP mice and WT mice. Genes colored in red represented dramatically upregulated genes, genes colored in green displayed remarkably downregulated genes, while insignificantly altered genes are colored in gray, log2 FC > 1, *Q* value < 0.05. **(F)** SLC7A11 of mouse lung parenchyma was stained by immunohistochemical (IHC) staining. **(G)** NCOA4 of mouse lung parenchyma was stained by IHC staining.

To quantify the gene expression profiles of CLP treatment, RNA sequencing (RNA-seq) was performed in lung tissues of CLP mice or sham group. Firstly, we created an overall heatmap of all differentially expressed genes (log2 fold change > 1, *Q* value < 0.05). Then, we picked out genes that had the most significant differences (top 50) and focused on several target genes. Hierarchical clustered heatmap identified a series of upregulated genes (*ALOX12*, *NCOA4*, *Fis1*, *YAP1*) linked to ferroptosis and mitochondria fission and mitigation of genes (*GPX4*, *SLC7A11*, *FTH1*) associated with antioxidative stress and anti-ferroptosis (**Figure 6A**). The Kyoto Encyclopedia of Genes and Genomes (KEGG) enrichment analysis was utilized to determine the most significantly altered signaling pathways involved in CLP-treated lung tissues, including the NF-κB signaling pathway and ferroptosis signaling pathway (**Figure 6B**). The protein–protein interaction (PPI) regulatory network analysis predicted a high clustering coefficient and potential interaction among several related genes, such as apoptosis, ferroptosis, and mitochondrial fission (**Figure 6C**). These enrichment genes regulated relevant signaling pathways. The network of KEGG pathways elucidated the potential crosstalk of these different signaling pathways (**Figure 6D**). The volcano plot also showed DEGs of CLP mice and the sham group (**Figure 6E**). Taken together, RNA-seq results indicated that YAP1 promoted ferroptosis in CLP mice lung tissue IHC staining showed that CLP stimulation reduced the accumulation of SLC7A11 (brown dye) in the lung tissue, while YAP1 knockout further diminished the content of SLC7A11 (**Figure 6F**). Moreover, we also observed that CLP stimulation gave rise to the accumulation of NCOA4 (brown dye), and YAP1 knockout exhibited more accumulation of NCOA4 (**Figure 6G**).

### YAP1 deficiency aggravated CLP-induced ferroptosis and ferritinophagy in lung tissue

**Figure 7:**
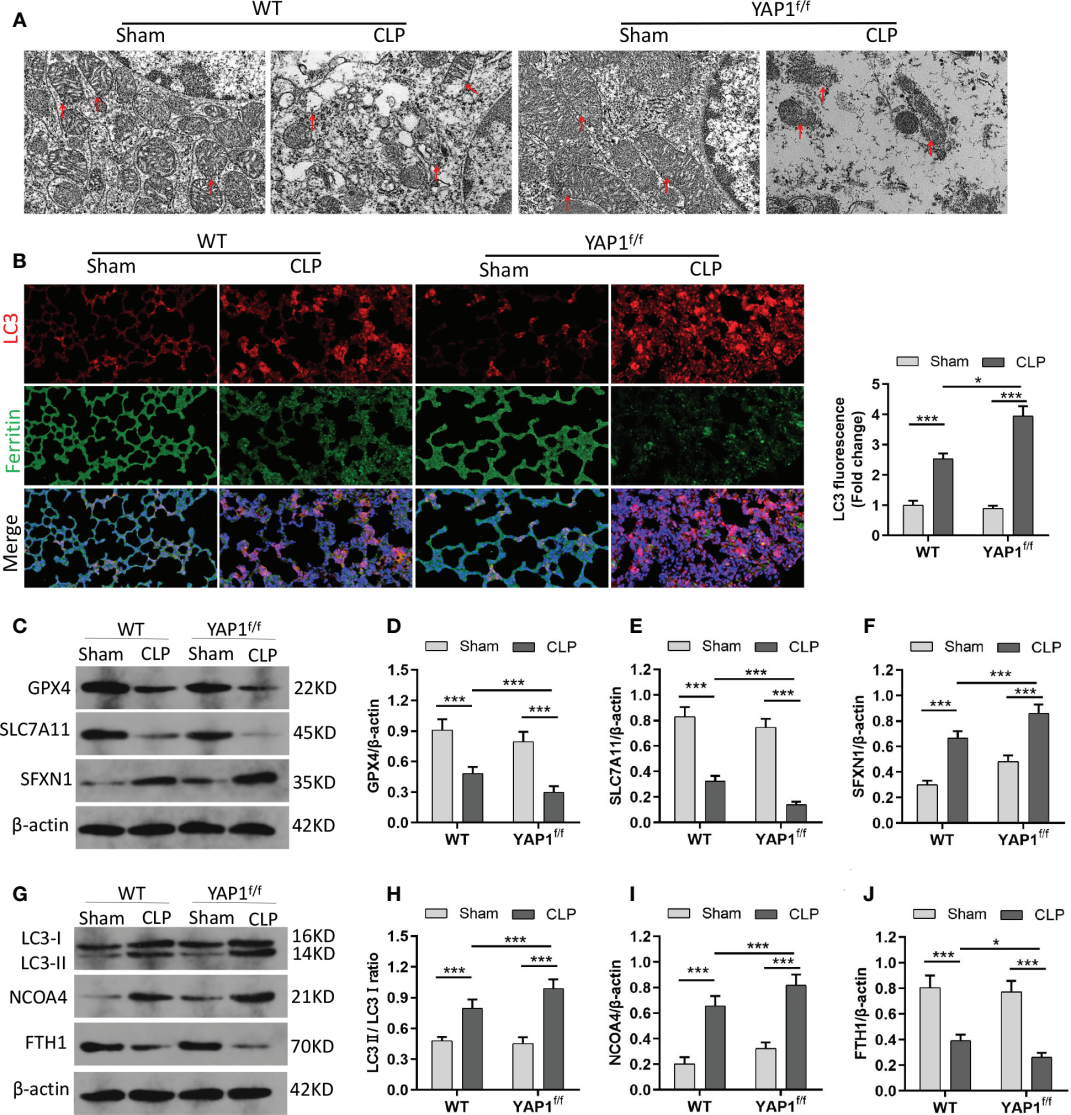
YAP1 deficiency aggravated CLP-induced ferroptosis and ferritinophagy in lung tissues. **(A)** Representative images shown by TEM. The red arrow indicates representative mitochondria in WT and YAP1^f/f^ mice lungs treated by CLP or not (*n* = 3 mice/group). **(B)** Fluorescence analysis showed representative images of ferritin colocalization (green) with tissue expression of LC3 (red) and quantification fluorescence intensity of LC3. Nuclei were counterstained by DAPI (blue). Light pink fluorescence represented the colocalization of ferritin and LC3. **(C–F)** Immunoblotting detection of the expression contents of GPX4 **(D)**, SLC7A11 **(E)**, and SFXN1 **(F)** in mice with indicated treatment. **(G–J)** Immunoblotting determination of the expression levels of LC3II/LC3I **(H)**, NCOA4 **(I)**, and FTH1 **(J)** in mice with equivalent treatment. Data are expressed as mean ± SD. *n* = 6; *p < 0.05, **p < 0.01, ***p < 0.001.

The morphological characteristics of ferroptosis were captured with TEM. The photographs revealed that the mitochondrial morphological structures were normal in WT mice and YAP^f/f^ mice without CLP injury. Following the CLP challenge, we observed significant aberrant mitochondria (red arrows in the WT+CLP group and the YAP^f/f^+CLP group), including reduction and even disappearance of mitochondria cristae and rupture of the mitochondrial outer membrane. Moreover, YAP1 deficiency further worsened CLP-induced mitochondrial damage (**Figure 7A**). In addition, the protein expression of GPX4 and SLC7A11 linked to ferroptosis and SFXN1 was determined. The data showed the significant upregulation of SFXN1 and downregulation of GPX4 and SLC7A11 during CLP treatment. However, these changes were further aggravated by YAP1 deficiency (**Figures 7C–F**).

Similar to the colocalization of LC3-GFP with ferritin in MLE-12 cells, we observed the fluorescent localization in CLP-treated mice with the presence or absence of YAP1 deficiency. As expected, YAP1 conditional knockout triggered the autophagy and promoted the degradation of ferritin in CLP-induced ALI (**Figure 7B**) (**Figures S4B, C**), indicating that YAP1 might affect ferroptosis by mediating ferritinophagy in sepsis-induced ALI. Western blotting showed that CLP treatment caused increases in LC3II/I ratio and NCOA4 levels and a decline in FTH1 levels. When mice experienced YAP1 conditional deletion, the LC3II/I ratio and NCOA4 climbed to higher levels, and FTH1 presented massive degradation (**Figures 7G–J**). In summary, our results suggested that the absence of YAP1 was able to aggravate ferroptosis and ferritinophagy in CLP-operated mice.

## Discussion

In this study, we unveiled that ferroptosis in pulmonary epithelial cells was generated in sepsis-induced ALI, and YAP1 may be a feasible protective agent that prevents ALI by inhibiting ferroptosis.

To explore the potential regulated mechanism of sepsis-induced ALI, we first performed the RNA sequence analysis. We discovered that the ferroptosis-related pathway was closely linked to the differentially expressed genes, and YAP1 was upregulated in sepsis-induced ALI models. Based on these observations, we picked out the ferroptosis-related genes and YAP1 for further experiments.

Since the term ferroptosis was formally put forward in 2012, related research has experienced exponential growth in the past several years. Ferroptosis is featured by the surplus iron-dependent accumulation of lipid peroxidation, causing membrane damage and non-reversible cell death (29, 30). Multiple cellular metabolic events could regulate ferroptosis, for instance, redox homeostasis, iron load, mitochondrial function, and lipid metabolism, as well as disease-relevant signaling pathways. Abundant evidence indicated that ROS partook in the pathogenesis of septic cardiac or liver injury (28, 31). Intracellular overburden of ferrous iron can lead to the accumulation of lipid ROS, which makes an imbalance of redox in cells and cell death. This study determined the levels of oxidative stress marker and discovered increasing intracellular Fe^2+^ and lipid ROS in both the MLE-12 cells and lung tissues after stimulation with LPS or CLP.

YAP1, which is an important transcription factor in the Hippo pathway, has been studied extensively and regarded as a potential approach for the onset mechanism of various diseases, such as cancer, atherosclerosis, fibrosis, and inflammation. Currently, YAP1 has been proven to promote alveolar epithelial regeneration and repair in a growing number of studies. The nuclear localization of YAP1 is pervasively distributed in pulmonary structural cells like lung epithelial cells. Homozygous deletion of YAP1 resulted in no new lung bud production, while selective loss of YAP1 disrupted lung branching morphogenesis and reduced cell proliferation (32). YAP could abolish inflammation and regulate pulmonary endothelial cell activation through preventing TRAF6-mediated NF-κB activation (33). In mice with bacterial pneumonia, YAP/TAZ deficiency in epithelial type II cells exhibited long-term inflammatory responses and tardive alveolar epithelial regeneration after lung injury (34). Furthermore, YAP1/TAZ mutant expression decreased the production of surfactant protein C (SPC), which is a representative feature of ALI/ARDS (14). Apart from the role of YAP1 in lung tissue, YAP activation reduced congenital inflammatory response to oxidative stress and necrosis/apoptosis to prevent the liver from ischemia–reperfusion (IR) stress (35). Nuclear YAP1 drove intestinal epithelial cell proliferation, which could cause post-inflammatory epithelial regeneration in ulcerative colitis (36). Hence, considering the protective role of YAP1 in promoting cell proliferation, we explored whether sepsis was a vital process in facilitating the ferroptosis of epithelial cells, which could be concerned with YAP1 activity. Our *in-vivo* study found that YAP1 deficiency aggravated sepsis-induced lung pathological damage and produced Fe^2+^ overload and higher MDA levels as well as more ROS accumulation in YAP1^f/f^ mice after CLP. Concurrently, YAP1 overexpression exhibited an anti-ferroptotic ability, as presented by the reduction in ROS accumulation and suppression of lipid peroxidation *in vitro*. These outcomes reminded us that ferroptosis participates in the genesis and development of sepsis-induced ALI, and YAP1 modulates the process by controlling Fe^2+^ and lipid peroxidation, which might provide a targeted strategy for mitigating sepsis-induced ALI.

Then, we detected other ferroptosis-related regulators. SLC7A11 and GPX4 were downregulated, while MDA and iron accumulation were upregulated in the LPS-treated human bronchial epithelial cell line (37), indicating that ferroptosis was on the front burner in LPS-induced ALI. ACSL4 is also necessary for facilitating ferroptosis and can insert unsaturated arachidonic acid into the cellular membrane, which produces a mass of lipid ROS. Pharmacological inhibition of ACSL4 can prevent ferroptosis-related diseases (38). In doxorubicin-induced myocardial damage models, YAP could decrease the expression level of ACSL4 (22). In lung adenocarcinoma, YAP diminished intracellular iron levels by promoting FTL transcription through transcription factor CP2 (TRCP2) (39). To clarify the mechanism of ferroptosis in sepsis-induced ALI, a transcriptome study of RNA sequencing was carried out, suggesting that the ferroptosis signaling pathway was involved in the pathogenesis of sepsis-induced ALI. Subsequently, our findings revealed that YAP1 knockdown attenuated the expression of GPX4 and SLC7A11 in CLP-treated mice, and YAP1 overexpression enhanced the expression of GPX4 *in vitro* and *in vivo*. YAP1 overexpression limited the expression of ACSL4 in LPS-treated cells. Briefly, YAP1 played a pivotal role in ferroptosis suppression *via* regulating the ferroptosis mediator in sepsis-induced ALI.

The mitochondria are the main sites of energy metabolism and the apoptotic targets of excessive ROS. Mitochondrial double membrane is the ideal site for ferroptosis. Increased mitochondrial ROS could trigger ferritinophagy and elevate the content of intracellular iron, eventually leading to ferroptosis (20). SFXN1 contributes to iron transportation into the mitochondria and promotes the utilization of erythroid mitochondrial iron. Furthermore, previous research discovered that SFXN1 is closely related to pathologic mitochondrial iron accumulation, which led to sideroblastic anemia (26). In YAP1^f/f^ septic mice, we detected more serious mitochondria damage and a higher expression level of SFXN1. Inversely, YAP1 overexpression blocked the expression of SFXN1 *in vitro* and allayed the generation of mitochondrial ROS.

Previous findings indicated that suppression of autophagy alleviated ferroptosis (19, 40). The catabolic process of autophagy involves the continuous degradation of substrates in lysosomes for maintaining cell homeostasis in response to hypoxia and stress as well as sepsis attack. Along with the occurrence of ferroptosis, autophagy is motivated, causing the consequent degradation of ferritin *via* NCOA4–FTH1 binding (41). NCOA4 mediated the autophagic degradation of ferritin in lysosomes; thus, cellular free iron is released to initiate the Fenton reaction. These cascades of reactions could be blocked by NCOA4 inhibition or autophagy disrupting, hence abrogating the accumulation of ferrous iron and ROS due to ferroptosis (42). Fang et al. (43) observed that compound 9a diminished the colocalization of Fe^2+^ in lysosomes and inhibited the NCOA4–FTH1 reaction. Qi et al. (44) clarified that curcumol could inhibit NCOA4 regulation of ferritinophagy to prevent hepatocyte senescence through the promotion of YAP. Zhang et al. (45) noted that YAP suppression might sensitize ferroptosis by inhibiting FTH1 in lung adenocarcinoma. In our experiments, the colocalization of ferritin and autophagosomes was observed by confocal microscopic evaluation, reflecting that YAP1 overexpression decreased the release of ferrous iron from lysosomes and lysosomal ferritin degradation. Moreover, we also found that YAP1 could inhibit the NCOA4–FTH1 interaction by co-IP assays, which was consistent with a previous report (45). An amount of Fe^2+^ from ferritin degradation stimulates SFXN1 production in the mitochondrial membrane, which subsequently transports Fe^2+^ into the mitochondria, triggering the cascade of mitochondrial ROS and eventually contributing to ferroptosis (28). SFXN1-induced mitochondria iron accumulation might be caused by NCOA4-mediated ferritinophagy in apelin-13 myocardiopathy (27). Our current observation supports the theory that YAP1 is linked to the NCOA4–FTH1 interaction. This interaction may affect SFXN1-regulated mitochondrial iron overload and mitochondrial ROS production, ultimately generating epithelial cell ferroptosis. Taken together, these experimental data suggested that YAP1 could suppress FTH1 autophagic degradation mediated by NCOA4, which was crucial for protecting pulmonary epithelial cells from sepsis-induced ferroptosis. Sepsis-induced ALI in mice was probably connected to ferritinophagy-mediated ferroptosis activation.

**Figure 8:**
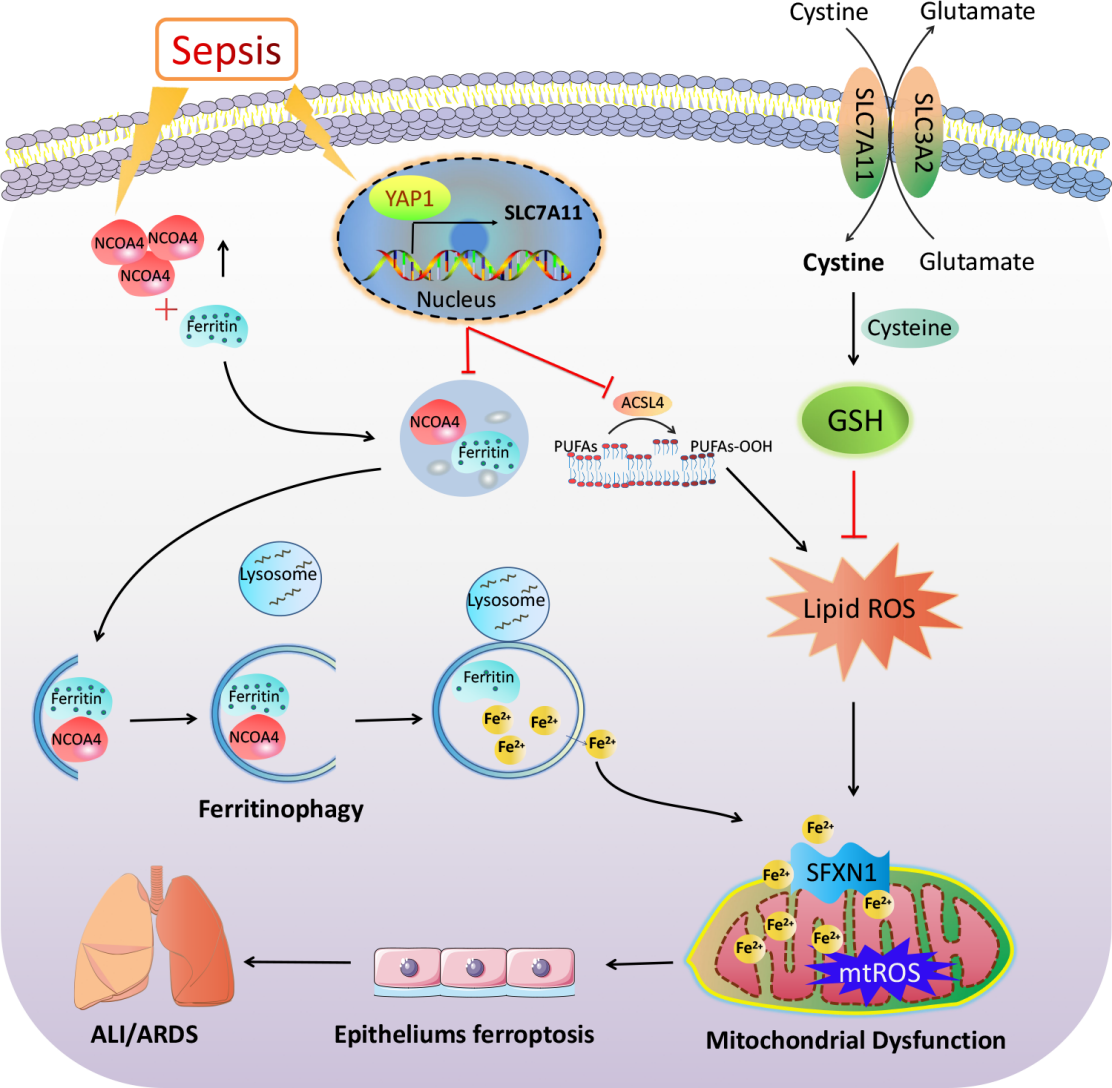
The mechanism illustration on the involvement of YAP1 in ferritinophagy and ferroptosis in sepsis-induced acute lung injury. Sepsis induces increased intracellular NCOA4 expression, which then has an interaction with ferritin and brings about the autophagic degradation of ferritin. YAP1 can suppress the degradation of ferritin into autophagosomes *via* inhibiting NCOA4-mediated ferritinophagy, then prevent ferrous iron from transporting into the mitochondria. YAP1 attenuates the accumulation of mitochondrial reactive oxygen species and lipid peroxidation and contributes to protecting mitochondrial function and repressing ferroptosis. Therefore, we conclude that YAP1 is involved in sepsis-induced acute lung injury by regulating the process of ferritinophagy-mediated ferroptosis.

In conclusion, our results demonstrate the important participation of ferroptosis and YAP1 in the pathophysiology of sepsis-induced ALI. YAP1 disrupted the NCOA4–FTH1 reaction and inhibited NCOA4-mediated ferritinophagy to prevent ferroptosis and subsequent mitochondrial ROS-related dysfunction in septic lung injury (**Figure 8**). These previous publications raise the possibility that YAP1 may be a protective ingredient of ALI/ARDS, and sustaining the expression of YAP1 could become an emerging therapeutic approach. Notably, elevated YAP levels have been formally confirmed to be related to cancer development. Consequently, one of the future challenges toward the application of YAP1 in lung diseases is activating YAP1 precisely without inducing cancer. Further investigation should be expected to explore the accurate regulation of YAP1 in sepsis-induced pulmonary diseases targeting ferroptosis.

## Data availability statement

The data presented in the study are deposited in the SRA (Sequence Read Archive) repository, accession number PRJNA836168.

## Ethics statement

The animal study was reviewed and approved by the Animal Ethics Committee of Wuhan University (No. 2021187).

## Author contributions

XL and XS conceptualized and designed the study. JZ, YZ, and YW performed the study and acquired the data. JW and AS analyzed the data. JZ drafted the manuscript. XL and XS revised the paper. All authors read and approved the final manuscript. All authors contributed to the article and approved the submitted version.

## Conflict of interest

The authors declare that the research was conducted in the absence of any commercial or financial relationships that could be construed as a potential conflict of interest.

## Publisher’s note

All claims expressed in this article are solely those of the authors and do not necessarily represent those of their affiliated organizations, or those of the publisher, the editors and the reviewers. Any product that may be evaluated in this article, or claim that may be made by its manufacturer, is not guaranteed or endorsed by the publisher.
