# Supplementary material for: YAP1 alleviates sepsis-induced acute lung injury via inhibiting ferritinophagy-mediated ferroptosis
Source: Front Immunol. 2022 Aug 1;13:884362. doi: 10.3389/fimmu.2022.884362 (PMC9376389; doi:10.3389/fimmu.2022.884362)
Supplement: Supplementary file 1 [file DataSheet_1.docx]

**Supplementary Information**

**YAP1 alleviates sepsis-induced acute lung injury via inhibiting ferritinophagy-mediated ferroptosis**

Jing Zhang1†, Yongping Zheng1†, Yun Wang1†, Jin Wang1, Aming Sang1, Xuemin Song2*, Xinyi Li1*

1 Department of Anesthesiology, Zhongnan Hospital of Wuhan University, Wuhan, China

2 Research Centre of Anesthesiology and Critical Care Medicine, Department of Anesthesiology, Zhongnan Hospital of Wuhan University, Wuhan, China

To construct LPS-induced inflammation model in MLE-12 cells, we explored the appropriate concentration and treatment time of LPS. Firstly, MLE-12 cells were treated with different concentrations of LPS (0.5 μg/mL, 1 μg/mL, 2 μg/mL, 5 μg/mL) for 24 h. The CCK-8 kit (Sigma-Aldrich, 96992) was used for the quantitation of viable cell number. Results showed that compared with the control group, the number of cells was significantly reduced when LPS concentration was greater than 1 μg/mL, so 1 μg/mL was chosen as the appropriate concentration of LPS. Secondly, MLE-12 cells were treated with 1 μg/mL LPS for 6 h, 12 h, 24 h and 48 h, and the number of cells was detected. The results indicated that the number of cells was significantly decreased compared with the undamaged control group when the treatment time of 1 μg/mL LPS was 12 h or longer. To explore the effect of LPS on YAP1 protein expression, we detected the expression of YAP1 following LPS treatment. The results revealed that the expression of YAP1 increases in response to LPS in a time-dependent manner, reached a maximum at 24 h, and reduced to basal level at 48h. Based on the above results, 24 h was chosen as the optimum treatment time of LPS.


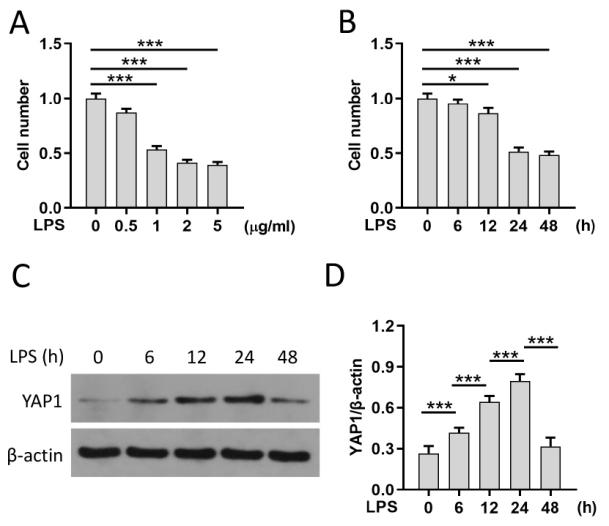


**Figure S1.** Preliminary experiments on the concentration and timing selection of LPS treatment in in vitro experiments. **(A)** MLE-12 cells were treated with LPS at different concentrations (0.5 μg/ml, 1 μg/ml, 2 μg/ml, 5 μg/ml) for 24 h, and the number of living cells was detected by CCK-8 kit. **(B)** MLE-12 cells were treated with 1 μg/mL LPS for different times (6 h, 12 h, 24 h, 48 h), and the number of living cells was detected by CCK-8 kit. **(C, D)** The expression of YAP1 after LPS treatment (1 μg/ml) at different times (6 h, 12 h, 24 h, 48 h) in MLE-12 cells were examined by Western Blot. *p < 0.05, **p < 0.01, ***p < 0.001.

To explore whether LPS induced the ferroptosis of pulmonary epithelial cells, we evaluated the effects of ferroptosis inhibitor and inducer on LPS-treated cells respectively. MLE-12 cells were divided into four groups: control group (Con), LPS group (LPS), LPS + Ferrostatin-1 group (LPS+Fer-1), and LPS+Erastin group (LPS+Era). Cells were cultured with 1 μM Ferrostatin-1 (Fer-1, SML0583; Sigma) or 3 μM erastin (E7781, Sigma) before LPS treatment. Ferrostatin-1 (Fer-1) was regarded as the effective inhibitor of ferroptosis, whereas erastin was an important promoter of ferroptosis that inducing cell death ^[1]^. Our results displayed that LPS stimulation induced significant cell death, while Fer-1 pretreatment remarkably mitigated the LPS-induced cell death and erastin pretreatment further aggravated the cell death. Likewise, MDA and Fe^2+^ assays showed that LPS stimulated distinct MDA upregulation and iron accumulation in MLE-12 cells. Fer-1 could inhibit the MDA upregulation and decrease iron accumulation in LPS-treated MLE-12 cells, while erastin produced adverse effects. Besides, Fer-1 restored the LPS-induced decrease in GSH contents to some extent, erastin pretreatment exhibited more obvious decline in GSH contents. These results indicated that ferroptosis was a vital form of LPS-induced cell death in MLE-12 cells.

1. Dixon SJ, Lemberg KM, Lamprecht MR, et al. Ferroptosis: an iron-dependent form of nonapoptotic cell death. *Cell*. 2012;149(5):1060-1072. doi:10.1016/j.cell.2012.03.042


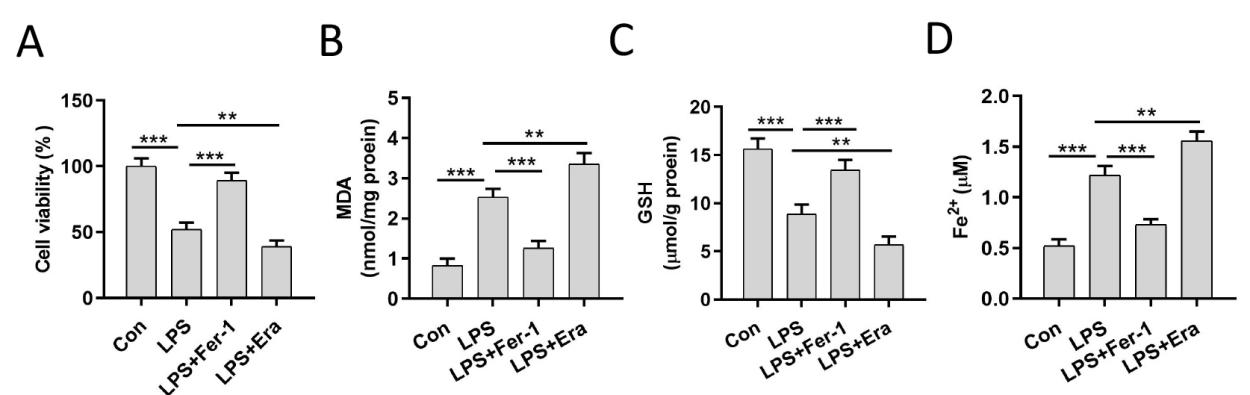


**Figure S2.** LPS (1 μg/ml for 24 h) stimulation induced ferropotosis in MLE-12 cells in preliminary experiments. **(A)** Cell viability was evaluated by CCK-8 kit. The contents of MDA (**B**), GSH (**C**), and Fe^2+^ (**D**) were detected by relevant kits. *p < 0.05, **p < 0.01, ***p < 0.001.

YAP is the main downstream effector molecule of Hippo signaling pathway, and can be directly phosphorylated by LAST1/2. Phosphorylated YAP (P-YAP) binds to the proteins and remains in the cytoplasm, then is ubiquitinated and degraded. P-YAP can not perform the function of promoting proliferation and inhibiting apoptosis. However, when YAP is transported into the nucleus, YAP can regulate tissue regeneration and maintain the balance between proliferation and apoptosis. The results showed that LPS promoted partial nuclei translocation of YAP1, Yap1 overexpression with LPS treatment significantly facilitated the nuclei translocation of YAP1.


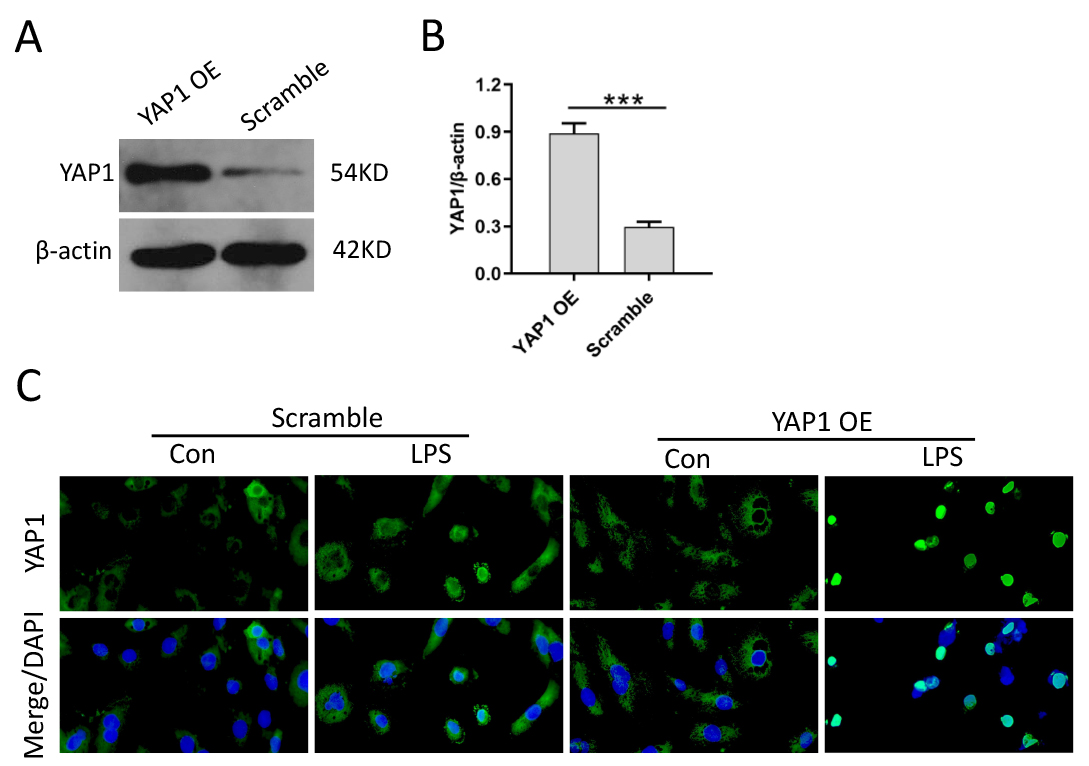


**Figure S3.** The verification of YAP1 overexpression and nuclear translocation of YAP1 were detected in MLE-12 cells. **(A, B)** Western blot analysis of YAP1 protein in the scramble group and YAP1 overexpression group. **(C)** The fluorescence images of YAP1 (green) of nuclear translocation in indicated groups. Nuclei were stained by DAPI (blue). ***p < 0.001.

**
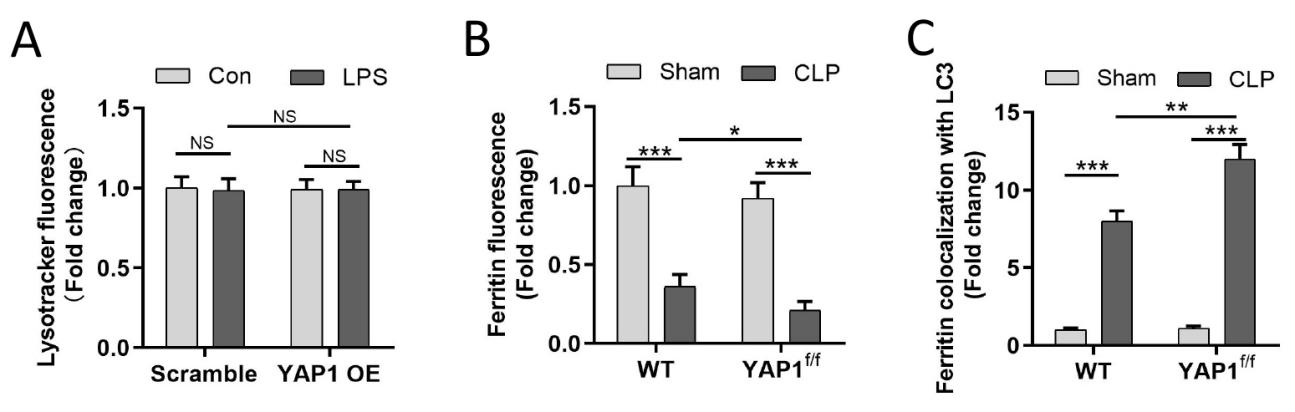
**

**Figure S4.** **(A)** Quantification of Lyso tracker staining in MLE-12 cells was showed. **(B)** Quantification of ferritin fluorescence in lung tissues was showed. **(C)** Quantification of ferritin colocalization with LC3 in lung tissues was showed. *p < 0.05, **p < 0.01, ***p < 0.001.
